# Describing the state of a research network: A mixed methods approach to network evaluation

**DOI:** 10.1093/reseval/rvac034

**Published:** 2022-10-28

**Authors:** James M Bowen, Mathieu Ouimet, Justin Lawarée, Joanna Bielecki, Ashley Rhéaume, Caylee Greenberg, Valeria E Rac

**Affiliations:** Program for Health System and Technology Evaluation, Ted Rogers Centre for Heart Research at Peter Munk Cardiac Centre, Toronto General Hospital Research Institute (TGHRI), Toronto, ON M5G 2C4, Canada; THETA Collaborative, University Health Network, 10th Floor Eaton North, 200 Elizabeth Street, Toronto, ON M5G 2C4, Canada; Health Technology Assessment and Network Analytics, Diabetes Action Canada, Toronto, ON M5G 2C4,Canada; Institute of Health Policy, Management and Evaluation, Dalla Lana School of Public Health, University of Toronto, Toronto, ON M5T 3M6,Canada; Department of Health Research Methods, Evidence and Impact, Faculty of Health Sciences, McMaster University, Hamilton, ON, Canada; Health Technology Assessment and Network Analytics, Diabetes Action Canada, Toronto, ON M5G 2C4,Canada; Département de Science Politique, Faculté des Science Social, Université Laval, Québec, QC G1V 0A6, Canada; Département de Science Politique, Faculté des Science Social, Université Laval, Québec, QC G1V 0A6, Canada; Program for Health System and Technology Evaluation, Ted Rogers Centre for Heart Research at Peter Munk Cardiac Centre, Toronto General Hospital Research Institute (TGHRI), Toronto, ON M5G 2C4, Canada; THETA Collaborative, University Health Network, 10th Floor Eaton North, 200 Elizabeth Street, Toronto, ON M5G 2C4, Canada; Département de Science Politique, Faculté des Science Social, Université Laval, Québec, QC G1V 0A6, Canada; Program for Health System and Technology Evaluation, Ted Rogers Centre for Heart Research at Peter Munk Cardiac Centre, Toronto General Hospital Research Institute (TGHRI), Toronto, ON M5G 2C4, Canada; THETA Collaborative, University Health Network, 10th Floor Eaton North, 200 Elizabeth Street, Toronto, ON M5G 2C4, Canada; Program for Health System and Technology Evaluation, Ted Rogers Centre for Heart Research at Peter Munk Cardiac Centre, Toronto General Hospital Research Institute (TGHRI), Toronto, ON M5G 2C4, Canada; THETA Collaborative, University Health Network, 10th Floor Eaton North, 200 Elizabeth Street, Toronto, ON M5G 2C4, Canada; Health Technology Assessment and Network Analytics, Diabetes Action Canada, Toronto, ON M5G 2C4,Canada; Institute of Health Policy, Management and Evaluation, Dalla Lana School of Public Health, University of Toronto, Toronto, ON M5T 3M6,Canada

**Keywords:** network evaluation, social network analysis, return on investment, research impact assessment, payback model, patient engagement

## Abstract

Diabetes Action Canada Strategy for Patient-Oriented Research (SPOR) Network in Chronic Disease was formed in 2016 and is funded primarily through the Canadian Institutes of Health Research (CIHR). We propose a novel mixed-methods approach to a network evaluation integrating the State of Network Evaluation framework and the Canadian Academy of Health Sciences (CAHS) preferred framework and indicators. We measure key network themes of connectivity, health and results, and impact and return on investment associated with health research networks. Our methods consist of a longitudinal cross-sectional network survey of members and social network analysis to examine Network Connectivity and assess the frequency of interactions, the topics discussed during them, and how networking effectively facilitates interactions and collaboration among members. Network Health will be evaluated through semistructured interviews, a membership survey inquiring about satisfaction and experience with the Network, and a review of documentary sources related to funding and infrastructure to evaluate Network Sustainability. Finally, we will examine Network Results and Impact using the CAHS preferred framework and indicators to measure returns on investment in health research across the five domains of the CAHS framework, which include: advancing knowledge, capacity building, informing decision making, health impact, and economic and social impact. Indicators will be assessed with various methods, including bibliometric analyses, review of relevant documentary sources (annual reports), member activities informing health and research policy, and Patient Partner involvement. The Network Evaluation will provide members and stakeholders with information for planning, improvements, and funding future Network endeavors.

## 1. Introduction

The establishment of networks facilitates collaborative initiatives and provides the opportunity for individuals with different backgrounds and capacities to work together toward a common goal. Networks can develop naturally over time or as a part of funded initiatives. By creating opportunities for socialization and collaboration, research networks can spawn interinstitutional and interdisciplinary relationships. Developing social relations based on trust increases the efficiency of information diffusion, facilitates knowledge creation, and prompts innovation (Griffith, Hu and Ryans 2000). A recent systematic review of health care networks has recommended that for those participating in networks, it is crucial to understand the network’s structure and characteristics, to attend to the function of the network, and finally to invest time into facilitating improvement (Cunningham et al. 2012a) leading to the conclusion that ‘nurturing networks should be encouraged’ (Ryan, Emond and Lamontagne 2014). Healthcare research networks can provide opportunities to solve unmet medical and health system needs by bringing together multiple perspectives to approach problems from a local, regional, national, or international context. With the public funding of healthcare networks over the past several decades, there is a societal need to evaluate the value of these collaborative relationships. The evaluation of networks is useful to not only the members of the networks themselves but also to the funders and other relevant stakeholders and beneficiaries of the networks’ output and impact. Ideally, evaluation of the network should be 1, imbedded into the performance measurement of the organization with awareness of the data required for evaluation (Cunningham et al. 2019); 2, be conducted longitudinally over the lifespan of the network (Fenton, Harvey and Sturt 2007; Cunningham et al. 2019); and 3, be adaptable to changes in the complexity and age of the network and 4, finally be reproducible with time.

**Figure 1.**
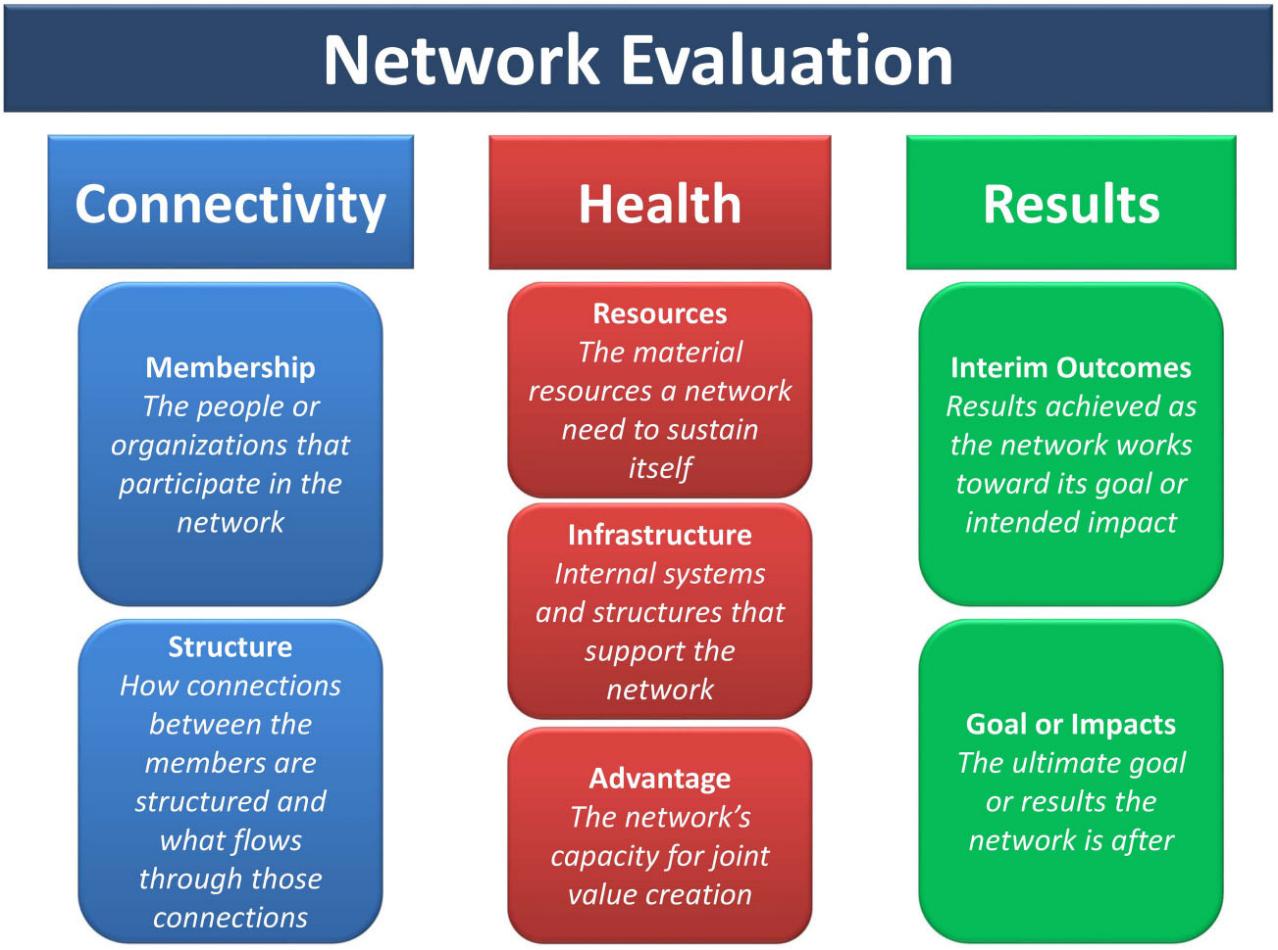
Three pillars of network evaluation (with permission Network Impact).

Network evaluation is complex since many aspects of the collaborative efforts of the members and institutions have to be considered, including the relationships between the network participants; the organization, governance, sustainability, and growth of the network; and finally, the results or output from the collaboration. Whether clinical or research-based, health network evaluation makes use of various methods, including social network analysis (SNA), by examining the interaction between members or actors associated with the network and through an examination of their relationships and collaboration (Long et al. 2014; Ryan, Emond and Lamontagne 2014; Long, Hibbert and Braithwaite 2016), qualitative semistructured interviews of members and stakeholders (Cunningham et al. 2012b; Leroy et al. 2017), examination of primary documentation associated with networks (Fenton, Harvey and Sturt 2007), and bibliometric analysis of publications (Leite et al. 2014). Several frameworks have also been proposed to account for various aspects of networks (Fenton, Harvey and Sturt 2007; Wixted and Holbrook 2012; Network Impact and Center for Evaluation Innovation 2014; Cunningham et al. 2019). Some of these frameworks are designed for networks in general, while others are specifically targeted toward health-related initiatives (Network Impact and Center for Evaluation Innovation 2014; Cunningham et al. 2019). From the Network Impact and Center for Evaluation Innovation, State of Network Evaluation, is a framework that encompasses three key pillars that examine a network's connectivity, health, and results (Figure 1; Network Impact (2022); Network Impact and Center for Evaluation Innovation 2014; Taylor, Whatley and Coffman 2015). Network Connectivity is an essential attribute for all networks. It is important to understand if the network effectively facilitates interaction among its members, which results in open and effective channels for active knowledge exchange and collaborative action and impact over time. Within this framework, connectivity has two dimensions: 1, membership (individuals and organizations); and, 2, structure (how connections between members are structured and what flows through those connections); Network Health within this framework reflects the network’s ability to sustain continuous enthusiasm, commitment, and engagement of its members to work together to achieve a shared vision, mission, and goals and covers three dimensions: 1, resources (external funding to sustain itself); 2, infrastructure (internal systems and structures that support the network—e.g. communication, processes, regulations); and, 3, advantages (capacity for joint value creation). Finally, Network Results, which are usually expressed as an overarching goal of achieving a particular change with a significant societal impact, have two dimensions: 1, interim outcomes that capture network performance and progress; and, 2, final intended goal/impact (Network Impact and Center for Evaluation Innovation 2014).

Measurement of the output or the results and impact of a health network can present challenges to examine the health research impact. Traditionally, the focus has been on documenting and measuring the academic output of healthcare research as the primary metric by where outcomes are evaluated. In January 2009, the Canadian Academy of Health Sciences (CAHS), sponsored by 23 different organizations across Canada, released a report entitled *Making an Impact—A Preferred Framework and Indicators to Measure Returns on Investment in Health Research*, which provides guidance for how returns on investment in health research can be examined using preferred indicators and metrics (Panel on Return on Investment in Health Research 2009). The framework is based on a ‘payback model’ or a ‘bottom-up’ approach developed by Buxton and Hanney in 1996, and consists of two elements: representation of the research process by a logic model and a categorization of the paybacks from activities (Buxton and Hanney 1996; Frank and Nason 2009; Donovan and Hanney 2011). The ‘payback model’ was initially adapted by the Canadian Institutes of Health Research (CIHR) in 2005, and then in 2008 following the CAHS initiative, in order to evaluate return on investment (ROI) in health and biomedical research (Panel on Return on Investment in Health Research 2009). The CAHS framework was developed to provide stakeholders, such as funders, with a preferred menu of indicators and metrics to assess ROI and measure the social, health, and economic benefits of investing in health research (Frank and Nason 2009; Panel on Return on Investment in Health Research 2009). Five main domains are covered within the framework to track health research impact: 1, advancing knowledge; 2, capacity building; 3, informing decision-making; 4, health benefits; and 5, broader economic benefit (Panel on Return on Investment in Health Research 2009). Across these domains, there are 66 indicators and metrics of research impact that can be examined (Frank and Nason 2009). The indicators of research activities are structured to apply to Biomedical Research, Clinical Research, Health Services Research, Population and Public Health Research, and Cross Pillar Research. The CAHS framework can be adapted to meet specific needs. It has been recently adapted by the Canadian Health Services and Policy Research Alliance (CHSPRA) to provide an enhanced approach to assess the impact of health services research and policy research specifically for informing decision-making (Canadian Health Services Policy Research Alliance (CHSPRA) Impact Analysis Working Group 2018). In Australia, the CAHS framework has been applied to examine the impact of research funded through the National Health and Medical Research Council (NHMRC) (Cohen et al. 2015) and has been used by the Association of Australian Medical Research Institutes (AAMRI) to develop indicators for examine research impact (Papageorgiou et al. 2021).

In this protocol, we propose a mixed-methods approach to conduct an evaluation of the Diabetes Action Canada Strategy for Patient-Oriented Research (SPOR) Chronic Disease Network. For this evaluation, we have integrated two existing frameworks related to network evaluation and health research impact, the State of Network Evaluation framework (Network Impact and Center for Evaluation Innovation 2014) and the Canadian Academy of Health Sciences impact framework (Panel on Return on Investment in Health Research 2009). The first examines three pillars of network connectivity, health, and results. In contrast, the second provides indicators and metrics that can be used to analyze results and evaluate health research returns on investment.

### 1.1 Setting

Canada’s SPOR is an initiative to engage patients and their families as proactive partners to shape healthcare research and health services. The SPOR is a ‘coalition of federal, provincial and territorial partners including patients and informal caregivers, researchers, health practitioners, policymakers, health authorities, academic institutions, charities and the private sector’ working toward a common goal to create ‘a collaborative, pan-Canadian process for identifying, establishing and addressing patient-oriented research priorities’ (Canadian Institutes of Health Research 2011, 2015). The CIHR currently fund research through seven SPOR Networks, five of which focus on chronic disease, one related to youth and adolescent mental health, and another studying primary and integrated health care innovations (Canadian Institutes of Health Research 2018). Diabetes Action Canada SPOR Network’s mission is *to develop patient- and research-informed innovations in equitable health care delivery designed to prevent diabetes and its related complications and to achieve the Quadruple Aim goals (improve: patient experience; population outcomes; health professional experience; health system cost)* with a vision to *transform the health trajectory for all Canadian men, women, and children with diabetes at risk for complications* (Diabetes Action Canada 2019).

Supported by funding from the CIHR for a 5-year grant beginning in 2016, our network membership initially consisted of 15 investigators from across Canada. Since that time, the Network has expanded to include Patient-Partners, Researchers, and Clinicians from across the country. Diabetes Action Canada SPOR Network’s membership consists of 99 researchers and 75 Patient Partners. Governing the Diabetes Action Canada SPOR Network is a Steering Council and Operations/Management Committee, Scientific Co-Leads supported by an Executive Director and administration team. The Patient-Partners, form an integral part of the Network and are organized into three (advisory) Circles: Collective Patient Circle; Indigenous Patients Circle; and Francophone and New Immigrants Circle.

Research activities of the Network are coordinated through Research Goal-Oriented Groups and Enabling programs. The Diabetes Action Canada SPOR Network’s efforts extend across three of the four pillars of Canadian Health Research (excluding basic biomedical sciences) to influence several areas of Canada’s health system and improve the health and well-being of Canadians economic and social prosperity. Initially, the activities of the Diabetes Action Canada SPOR Chronic Disease Network were organized into eight Goal Groups; however, as our Network has evolved and grown, additional groups/programs have been added. Currently, the Diabetes Action Canada SPOR Network is organized into The *Research Goal Oriented Groups* (*N* = 6) which are: 1, Diabetic Retinopathy Screening; 2, Indigenous Peoples Health; 3, Innovations in Type 1 Diabetes; 4, Digital Health for Diabetes Research and Care; 5, Foot-care to Prevent Amputations; and, 6, Aging Community and Population Health. In addition, there are *Enabling Programs* (*N* = 5), which are: 1, Patient Engagement; 2, Training and Mentoring; 3, Knowledge Translation; 4, Sex and Gender; and 5, Health Technology Assessment and Network Analytics. In the Fall of 2018, the decision was made by the Network to incorporate Health Technology Assessment and Network Analytics into the organization’s enabling programs. The role of the Network Analytics component of the Diabetes Action Canada SPOR Chronic Disease Network is to provide the network members and funders with an evaluation of the status and impact of the Network across the lifetime of the organization.

**Figure 2.**
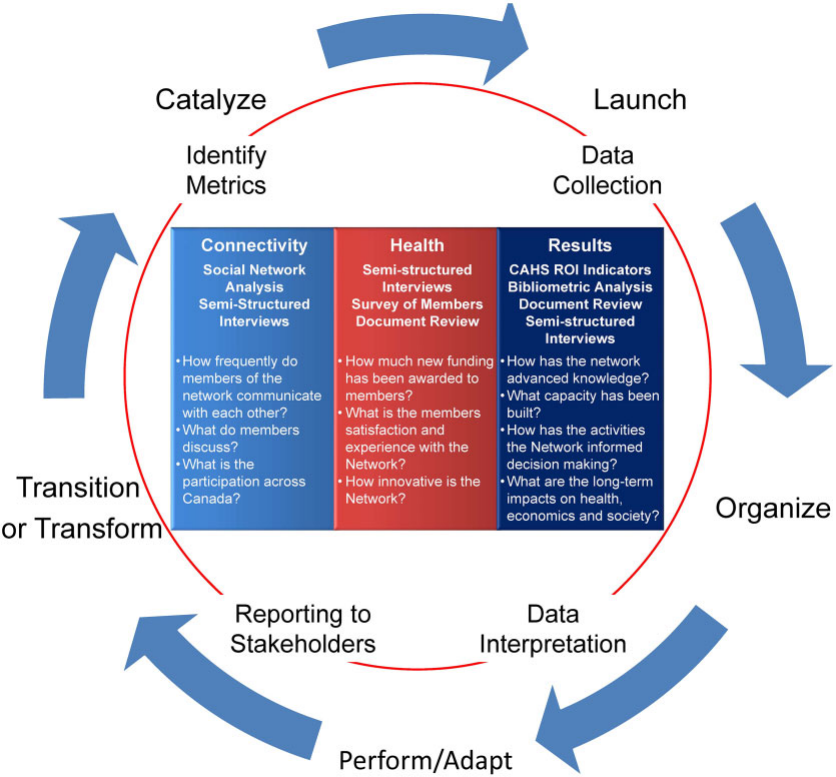
Network evaluation framework.

## 2. Rationale for evaluation

The rationale behind developing a such a process for longitudinal network evaluation is to provide stakeholders, both funders and Network members, with an analysis of Network membership, and their impression of the Network, document members’ activities and collaboration, and Network impact, to be accountable to funders for the resources provided, assist with the advancement of the network and quality improvement, and finally highlight successes of the Network to stakeholders to support the sustainability. Over the lifetime of the Network, we hypothesize that the Diabetes Action Canada SPOR Network, through its relationships and collaboration between members (Connectivity); supported by the structure of the Diabetes Action Canada SPOR Network, will result in continued research funding, expanded research capacity and patient partner engagement (Health); with impactful health research results, both academically, clinically and related to health policy development, and this will have a broader economic and social impact (Results) on the lives of those individuals living with chronic disease and Canadian Society as a whole.

## 3. Approach to network evaluation

The integration of the two frameworks was originally conceived as a part of a grant proposal related to the TRANSFORM HF Network (Rac 2018). The State of the Network Evaluation Framework was considered as the first component for the Network Evaluation to examine the life cycle of a network (Network Impact and Center for Evaluation Innovation 2014). This framework, however, does not provide details regarding measuring the results or outputs associated with the network activities, specifically related to health research and ROI. An updated systematic review examining the impact of health research, in the context of health technology assessment, was examined (Raftery et al. 2016). Within this review, the CAHS framework is discussed in relation to other proposed frameworks for examining the impact of health research and the CAHS framework was selected to be incorporated into our proposed Network Evaluation for the following reasons: 1, it provides a conceptualized list of metrics, 2, permits the ability to evaluate immediate and long-term returns on investment, 3, it is the suggested framework for evaluation of Canadian research enterprises, and had been adopted to measure the Results within the integrated frameworks (Frank and Nason 2009), 4, is based on the ‘Pay-Back’ logic model proposed by Buxton and Hanney (1996) which members of our group had previously used (de Oliveira et al. 2013), and 5, had been adopted internationally outside of the Canadian context. Furthermore, recognizing that the CAHS framework has been described as generic and adaptable (Panel on Evaluating CIMVHR 2012), we considered that the incorporation of the two frameworks provided a more comprehensive approach to meet the need to evaluate the Diabetes Action Canada SPOR Chronic Disease Network. The integration of the frameworks permits the evaluation of the Network over time using a wide variety of methods, and the capture of relevant metrics, and the long-term ROI associated with the network activities. Essential to this process is the proactive reporting of the Network Evaluation results to both internal and external stakeholders to guide the adaptation, transition, and transformation of the Network. This approach also provides a quality improvement process for the Network as it transitions through the stages of a network life cycle (Figure 2).

**Figure 3.**
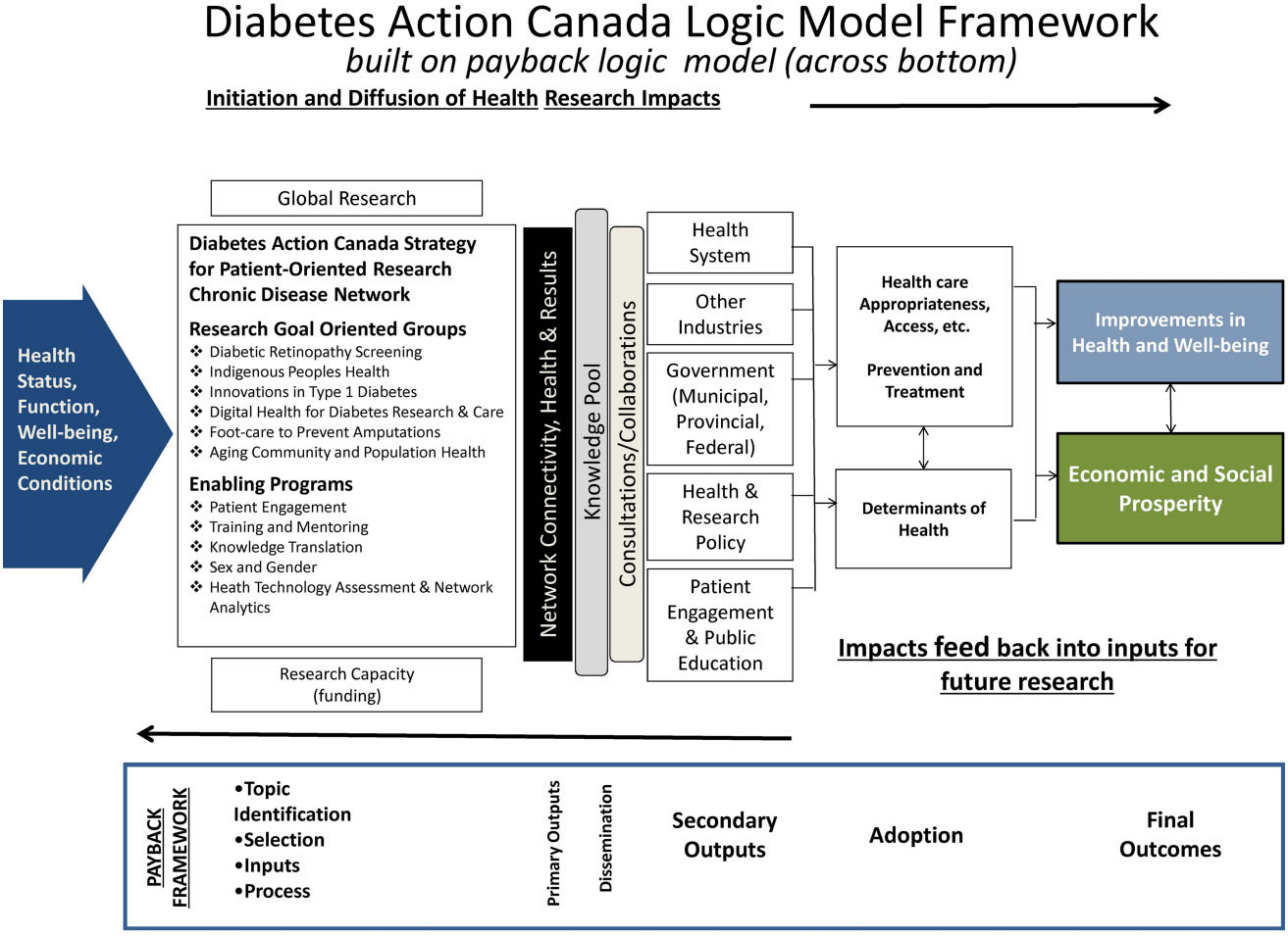
Diabetes Action Canada SPOR network logic model framework.

As a core component of the CAHS Framework, a logic model for the Diabetes Action Canada SPOR Network’s research activities was created (Figure 3). The inputs for the logic model are each of the Research Goal Oriented Groups and the Enabling Programs. Forming the first of the primary outputs in logic model is the resulting Network, with the interactions between the individual members and groups, the joint value of the relationships and collaboration to gain additional research funding, engagement with patient partners, and capacity building of the Network, which collectively represents the Connectivity, Health, and Results pillars in State of Network Evaluation framework (Figure 3; Network Impact and Center for Evaluation Innovation 2014). Thus, in this adapted framework, we consider many important aspects for the network evaluation including: 1, the relevance of the structure and function of the Network; 2, the number and diversity of actors coming from different stakeholder groups, and how they connect and view their roles; 3, the network’s continual emergence and evolution; 4, the timing to effectively develop and organize the network activities to demonstrate results with impact; and 5, the recognition of the ‘chain of impact’—the network’s impact on its members, the members’ impact on their local communities, and the members’ collective impact on their broader environment; and 6, the approach to evaluation that must be revisited periodically. Network Connectivity, Health, and the Network Results collectively support disseminating knowledge from the Network activities and facilitate further collaborations and consultations with stakeholders. Within the logic model, it is these activities that lead to secondary outputs impacting the health system, other industries, government at all levels, influencing health and research policy and in the case of the Diabetes Action Canada SPOR network, through participation of patient partners and their perspectives influence, enhance patient engagement and public education, and lead to broader health and well-being, and economic and societal prosperity. Networks typically move through stages of development, including catalyzing, launching, organizing, performing and adapting, and transitioning or transforming stages. We intend to complete the Network Evaluation up to a minimum of three times over the initial funding of the Diabetes Action Canada SPOR Network and provide to the executive, members, and stakeholders with reports outlining Connectivity, Health, and Results associated with the Network. This longitudinal evaluation examines assessable attributes and performance indicators, depending on the age of the network and the stage of the life cycle of the network. All components of the Network Evaluation will be presented to stakeholders to provide a holistic and complementary perspective of the Network. Findings from the semistructured interviews and questionnaires will be interpreted with the SNA, bibliometric analyses, and review of documentary sources.

Below we describe the proposed methodological approaches to evaluate the Diabetes Action Canada SPOR Chronic Disease Network using the State of Network Evaluation and CAHS Framework indicators. The network evaluation's SNA and semistructured interview portions were reviewed and approved by the Université Laval’s Research Ethics Board (*2018-336-A-1/08-05-2019*) and University Health Network’s Research Ethics Board (19-5773). All gathered data will be anonymized and stored according to the Board’s regulations. Members of the research team also signed a formal agreement to ensure that all data remained confidential.

## 4. Network connectivity

### 4.1 Membership and structure

The network connections between the members will be assessed longitudinally using SNA methods. A detailed description of the methods used is described elsewhere (Lawarée et al. 2022). Briefly, at each evaluation stage, a cross-sectional survey of the Networks’ members, regardless of status (e.g. patient partners, researchers, administrative employees, chair council members, co-leads, etc.), who are formally involved in the SPOR Network, will be conducted using Qualtrics’s web-based survey platform (Qualtrics, Provo, UT). Members will be identified through the Diabetes Action Canada SPOR Network’s administrative datasets within the previous fiscal year. The e-survey questionnaire includes two questions to measure the frequency of the relationships between Diabetes Action Canada SPOR Network’s members and the topics discussed during these exchanges. For each listed individual, participants are first asked if they have had contact with the listed member and to indicate the frequency of the relationship on the following 5-point scale: 1, daily, 2, weekly, 3, monthly, 4, quarterly, or 5, yearly. To examine the members’ regional distribution across Canada, their region or province of work is requested. Based on administrative data for the Diabetes Action Canada SPOR Chronic Disease Network, each actor is assigned to one of the following primary functions: research, patient partner, governance (e.g. Steering Council, sponsor partner) and administration (e.g. executive director, SPOR Network’s employees or project coordinators/managers). Participants are asked to indicate the topic(s) they discussed during their exchanges with each individual using the following categories (i.e. participants could tick all categories) that were adapted from published SNA of health care networks (Blanchet and James 2012; Chambers et al. 2012; Cunningham et al. 2012a): Scientific research; Training; Patient engagement; Management and operations; Governance and coordination; Commercialization of research findings; Transfer of research findings; and Other. The density and the centralization scores for each discussion topic will be evaluated. The cross-sectional network data will be analyzed with VOSviewer (van Eck and Waltman 2019) and Pajek (Batagelj and Mrvar 2018).

## 5. Network health

### 5.1 Resources, infrastructure, and advantage

We will review relevant internal and external documentary resources to examine the available resources, infrastructure, and advantages related to network health. We will explore aspects of infrastructure creation, including additional funding obtained through grants awarded to Diabetes Action Canada SPOR Network Investigators, whether as a Principal Investigator or Co-investigator and the advantages of creating the value beyond the individual and describing network sustainability.

The review of internal and external documentary resources is to further explore network health by assessing the initial resources provided by the CIHR, network governance structure, and membership, and capacity of the Network for joint value creation in terms of additional funding obtained by Diabetes Action Canada SPOR Network members and the creation of Network strategic relationships. Where necessary, clarification of the nature of the funding from the SPOR Network Investigators will also be obtained.

We will further explore the Network Health and the capacity for joint value creation through a cross-sectional network survey of the Diabetes Action Canada SPOR Network’s members. We will ask members about their satisfaction and experience with the Diabetes Action Canada SPOR Network over the past year. A 7-point Likert scale will be used to evaluate the member’s experience with the network. The members will be asked about their level of satisfaction with the Diabetes Action Canada SPOR Network’s products or services, the potential for achievement of overarching objectives (success), the ability for the network to compete with other formal health care-related research networks, and with management or leadership of the network. Lastly, the members will be asked how innovative they considered the Diabetes Action Canada SPOR Network relative to formal healthcare-related research networks.

We will then examine the perceived advantages of belonging to the Diabetes Action Canada SPOR Network and the capacity for joint value creation through semistructured interviews with Diabetes Action Canada SPOR Network members within the Network itself. We will also explore how the Diabetes Action Canada SPOR Network met members’ expectations and key perceived success and barrier factors for compelling interactions and collaborations within the Network. These semistructured interviews (primarily by phone) with a purposive sample of Diabetes Action Canada SPOR Network members from the different groups will be conducted. The interview guide has been closely modeled on the *CDC Evaluation Interview Guide*, which was developed to evaluate the impact of specific communities on individual members, organizations, and public health (Centre for Disease Control and Prevention 2015). The interview guide includes 18 open-ended questions (Supplementary Appendix) designed to achieve the following objectives:

- to describe the perceived purpose of the Diabetes Action Canada SPOR Network from the members’ perspective;
- to assess the extent to which the Diabetes Action Canada SPOR Network met member expectations;
- to describe outcomes associated with the Diabetes Action Canada SPOR Network;
- to identify key success factors for effective interactions and collaborations within the Diabetes Action Canada SPOR Network; and,
- to identify perceived barriers or other factors that limited interactions and collaborations within the Diabetes Action Canada SPOR Network.

All interviews will be recorded and transcribed verbatim. NVivo 12 will be used to analyze the data using a thematic analysis approach based on a predefined codebook.

## 6. Network results

We plan to evaluate the Diabetes Action Canada SPOR Network using the CAHS framework indicators when considering the Network Results. For this step, we independently assessed (JMB and VER) each of the 66 indicators to determine their relevance for evaluating the Network. Each of the indicators was itemized in tabular format and assessed for 1, relevance to the Network, 2, the proposed methods for data collection, 3, the feasibility of collecting and analyzing the data, 4, the appropriateness of the indicator based on the age and maturity of the Network, its research activities, and 5, consideration of frame of reference as outlined in the framework. Disagreements were then resolved by consensus. Where possible, ambivalence related to the indicators, we consulted relevant stakeholders to clarify the use of the metric. For the initial evaluation, the domains and associated indicators that we will examine are advancing knowledge, capacity building, and informing decision-making (Table 1) which are primary and secondary outputs, or proximal outputs as described by Graham et al. (2018) in the logic model (Figure 3). Some relevant indicators are to be excluded from the initial evaluation and deferred for future analysis, due to lack of maturity of the Network and the early nature of research projects and are to be assessed for inclusion for future iterations of the Network Evaluation. These indicators were primarily matched with the State of Network Evaluation Dimension of Goals and Impacts and the impact of the Network activities concerning health and broader social and economic outcomes (Table 2). Outlined below are the proposed approaches to be considered to provide data for each of the indicators.

**Table 1.** Network results—interim outcomes and Canadian Academy of Health Sciences framework impact and indicators.

State of network evaluation dimension	CAHS framework impact	CAHS category	Indicators (*N* = 36)	Proposed methodology
Interim Outcomes	Advancing Knowledge	Quality	Relative citation impact	Bibliometric analysis
			Highly cited publications	
			Publications in high-quality outlets (or desired outlets)	
		Activity	Share of publications	
			Publication counts	
		Outreach	Field analysis of citations	
			Coauthor analysis	Bibliometric Social Network Analysis
		Contextual/Structural	Relative activity index	Bibliometric analysis
		Aspirational Indicators	Expanded relative citation index	
			Relative download rate	
			Research diffusion	
	Capacity Building	Personnel	Graduated research students in health-related subjects	Review of Annual Reports and Metrics
			Numbers of research and research-related staff in Canada	
		Funding	Levels of additional research funding	
		Infrastructure	Infrastructure grants ($)	
			Percent of activity grants with infrastructure support	
		Aspirational Indicators	Receptor capacity	
			Absorptive capacity	
	Informing Decision Making	Health Related	Health Care—Use of research in guidelines	Bibliometric analysis and reporting/survey of members
			Public Health—Survey of public health policy makers	
			Social Care—Use of research in guidelines	
			Other—Researcher reported use of findings outside health	
			Health Related Education—Research cited in ongoing health professional education material	
		Research	Research Funding—Citation analysis of successful funding applications	Bibliometric analysis and reporting/survey of members
			Research Policy—Consulting to policy	
			Research Policy—Requests for research to support policy	
			Research Education—Research used in curricula for new researchers	
		Health Products Industry	Number of patents licensed	Bibliometric analysis and reporting/survey of members
			Clustering/co‐location	
			Consulting to industry	
			Collaboration with industry	
			Use of research in stage reports by industry	
		General Public	Advocacy Groups—Research cited in advocacy publications	Annual Reporting of KT activities and Workshops
			Public Education—Public lectures given	
		Aspirational Indicators	Media—Media citation analysis	Grey Literature review and annual reporting by Members
			Public Policy Use—Citations in public policy documents	

**Table 2.** Network results—goal or impact and Canadian Academy of Health Sciences framework impact and indicators.

State of network evaluation dimension	CAHS framework impact	CAHS category	Indicators (*N* = 30)	Proposed methodology
Goal or Impacts	Health	Health Status	Morbidity to include functional impacts—Prevalence	Evaluation of individual studies and incorporation into protocols where relevant
			Morbidity to include functional impacts—Incidence	
			Mortality—Potential Years Life Lost	
			Quality Adjusted Mortality—Quality Adjusted Life Years (QALYs)	
			Quality Adjusted Mortality—PROMs	
		Determinants of Health	Modifiable Risk Factors—Example: obesity; alcohol consumption	Evaluation of individual studies and incorporation into protocols where relevant
			Social Determinants—Example: education levels; social cohesion	
			Environmental Determinants—Example: air pollution levels	
			Acceptability—Example: self‐reported patient satisfaction	
			Accessibility—Example: wait times	
			Accessibility—Example: appointment statistics	
			Appropriateness—Example: adherence to clinical guidelines	
			Competence—Civil lawsuits against health system	
			Continuity—Self‐reported continuity of care	
			Effectiveness—Example: re‐admission rates	
			Efficiency—Actual vs. expected hospital stay	
			Efficiency—Cost input versus output	
			Safety—Example: adverse drug effects	
			Safety—Example: hospital-acquired infections	
	Broad Economic and Social	Activity Impacts	Economic rent (Labor rents)	Future Consideration pending Network evolution
		Commercialization	Licensing Returns ($)	
			Product Sales revenues ($)	
			Valuation of Spin-Out Companies ($)	
			Economic rent (Producer rent and spillover effects)	
		Health Benefit	Health Benefit in QALYs per health care dollar	
			Health Benefit in PROMs per health care dollar	
		Well-Being	Annual report of HRSDC	
			Happiness	
			Level of Social Isolation	
		Social Benefit	Socioeconomic Status	

### 6.1 Advancing knowledge

We will examine the interim results associated with the Network through a bibliometric analysis to evaluate the impact on advancing knowledge and the associated indicators (Panel on Return on Investment in Health Research 2009; Panel on Evaluating CIMVHR 2012). Diabetes Action Canada SPOR Network requests, as a component of their annual reporting to CIHR, that the members submit the citations of papers that have been published as a part of their Network activities. We will use these reported citations as the foundation for the bibliometric analysis. Citation identification and data cleaning as described by Bender et al. (2015) will be employed. Validation of the coauthors, year of publication, using a combination of information from medical literature citation databases such as PubMed, Embase, SCOPUS, and Google Scholar, will be completed. To examine collaboration across Canada, we will compute additional bibliometric and demographic information from full-text publications, including the number of coauthors, the author's province, the involvement of coauthors from government or the private sector and the frequency and country of residence of international authors. With the support of an academic librarian, targeted literature searches in Google Scholar or SCOPUS will be conducted to identify publications stating funding from Network that members did not declare to the administrative body of the network. For the coauthor SNA, we will use a combination of VOSviewer (van Eck and Waltman 2019) and Pajek (Batagelj and Mrvar 2018) SNA software to analyze and illustrate the Diabetes Action Canada SPOR Network coauthor publication interactions. Using either SCOPUS, Web of Science, or other bibliometric citation platforms, we will also compute publication and citation count, average citation counts and range, benchmarking metrics, field analysis for study topics, and proportion of papers within each field. All abstracted data will be managed in Excel and Endnote.

### 6.2 Capacity building

To evaluate the ability of the Diabetes Action Canada SPOR Network to build research capacity related to the training of new researchers and students and the employment of research staff, we will review the annual reporting and funding for undergraduate and graduate students, as well as postdoctoral fellows. The employment of research staff by each program will be obtained from the investigators and administrative staff of the SPOR Network. Throughout the Network, the total number of students completing their training will also be reported.

Information regarding new funding awards will be obtained through Network Annual Reporting before each evaluation to determine the incremental funding received since the Network's inception or last Network Evaluation. We will then get further information about the identified grants reported by investigators through the CIHR Funding Decisions Database (Canadian Institutes of Health Research 2021a) and the Canadian Research Information System (Canadian Institutes of Health Research 2021b). Funding from granting agencies other than CIHR reported by the Investigators will be captured. To further understand the collaboration between investigators and the capacity building of Network investigators, information on the funding will be abstracted from the above databases and published papers, the bibliometric analysis will be conducted with VOSviewer (van Eck and Waltman 2019) and Pajek (Batagelj and Mrvar 2018). This analysis can also be used to examine the successful funding application indicator.

### 6.3 Informing decision-making

Two of the CAHS indicators involve examining the use of research within health, service, and social care services guidelines. To evaluate these indicators, clinical, service, or social care guidelines that subsequently cite the articles published by Network members will be identified as a bibliometric analysis component. Furthermore, the CAHS framework outlines indicators related to informing decision-making and public health policy and consulting to policymakers as secondary outputs in the logic model. Within the scope of the Diabetes Action Canada SPOR Network, we considered that these indicators should be expanded to include consulting to research policy and health policy. To evaluate this indicator, we will examine the meetings with various levels of government with the Diabetes Action Canada SPOR Network for all members. We will survey members, including the Principal Investigators, Co-Investigators, Leads and Co-leads of the program, and the Executive Director. Examination of influence on decision-making at the local, provincial, national, and international levels of government will be captured to understand the level of health policy influence.

Furthermore, academic and industry collaboration, consultation and clustering/colocation are CAHS indicators that can be evaluated in relation to the activities of the SPOR Network. The Diabetes Action Canada SPOR Network engages in academic-corporate collaboration through funding support, industry partners as collaborators on projects, and guidance through the Steering Committee. We will examine this engagement through bibliometric analysis of full-text publications for coauthorship and funding or material support reporting. The Diabetes Action Canada SPOR members are also asked to report on annual basis funding sources for projects. These annual reports will also be examined. As the network matures, commercialization opportunities may arise and will be captured as a component of the evaluation.

The Network, as a component of its annual reporting, requests that its members provide information about media articles and interviews. Although listed as an aspirational indicator within the CAHS framework related to mentions in newspapers, we have expanded this to include interviews by radio, television, and podcasts as a suitable medium for communicating clinical and research findings and Network activities to the general public. For the final aspirational indicator of citations in public policy documents, grey literature searches within Google Scholar will be completed.

### 6.4 Health and broad economic and social impact

Based on the stage of development and maturity of the Network, with many of the projects yet to be completed, we considered that the ability of the Network activities to have measurable health impacts would be limited at this time. Upon careful review of the indicators outlined within the domain of health impact, we propose that the outcomes of the Diabetes Action Canada SPOR Network should be evaluated on a per-project basis to identify changes in health status or determinants of health (Table 2). The broader implementation of programs developed through research activities of the Diabetes Action Canada SPOR Network members can contribute to Canadians' overall improvement of health. To assess the Health Impact domain within the CAHS framework, health status and determinants of health-related indicators will need to be incorporated *a priori* into the project protocols and activities. Each project will require an assessment of the outcomes related to the Network activity. We have also considered that each Research Goal Oriented Groups publications and research activity can be examined in their entirety to examine potential health and policy impact.

Similarly, we considered that the ability to assess the impact of the Diabetes Action Canada SPOR Network on broader economic and social aspects in Canada is more appropriate for later stages of the evaluation to capture the longer-term outcomes. The ability of the network to have an impact on commercialization, health benefit, well-being, and social benefit may be possible. However, these indicators will need to be inferred from the specific network activities and projects.

### 6.5 Patient partners

As this is a Patient-Oriented Research Network, we have considered that the two CAHS indicators of Advocacy Groups and Public Education regarding the impact of research on the General Public do not fully provide the ability to examine the impact of the Patient-Partners on the Network. A broader scope of indicators was considered necessary to describe the involvement of the Patient-Partners and their influence concerning Network activities. We will examine, through annual reporting, the participation of the Patient-Partners in educational workshops, as participants and leaders, coauthors on grant submissions and publications, network committees and patient circles, and other network-related activities.

## 7. Mixed-methods approach

A convergent parallel mixed-methods design (Creswell and Piano Clark 2017) will be used to interpret the findings from the various qualitative and quantitative methods within each of the three domains of Network Connectivity, Health, and Results. The qualitative and quantitative research methods and data collection will be conducted at the same time for each cross-sectional Network Evaluation. Each domain of the Network Evaluation will have equal emphasis providing a broad perspective of the Network. Four major steps will be employed for this convergent parallel mixed-methods study (Creswell and Piano Clark 2017). First, we will collect quantitative and qualitative data for each of the domains in a concurrent but separate manner. In the second step, we will analyze the data resulting from the SNA, semistructured interviews, review of documentary sources and bibliometric results and SNA independently following traditional quantitative and qualitative analytical approaches. After we produce the initial results, we will merge the results in a third step. Some of the merging strategies that we may use include direct comparison of the quantitative and qualitative results in a table/matrix format or transformation of the results (e.g. quantitative results into themes) to support the ability to interpret the results in further analysis (Creswell and Piano Clark 2017). In the fourth and final step, we will interpret the merged results to explore to what extent and in what ways the Network Connectivity, Health, and Results from quantitative and qualitative studies converge, diverge from each other, relate to each other, and/or combine to create a deeper and holistic understanding of the Diabetes Action Canada SPOR Network.

## 8. Discussion

Research network evaluation is not entirely new to the Canadian health research setting. Some previous evaluations of Network Centres of Excellence of Canada (NCE’s) have been conducted (Wixted and Holbrook 2012; Networks of Centres of Excellence 2017). Here, we present a way to incorporate a recommended Canadian framework for ROI related to health research (Panel on Return on Investment in Health Research 2009) with a framework used for evaluating networks which examines the connectivity, health, and results associated with networks (Network Impact and Center for Evaluation Innovation 2014). The choice of these two frameworks was initially proposed for the longitudinal evaluation of another network by one of the authors. The State of the Network Evaluation framework provides a structure to examine network activities, however, it is limited in specific indicators related to health research within the CAHS framework. Integrating the two evaluation frameworks to examine the Diabetes Action Canada SPOR network is a unique approach to permit the comprehensive and longitudinal measurement of the network activity. Although some of the indicators within the CAHS framework could also be associated with Network Connectivity or Health, for practical purposes, all indicators were integrated with the Network Results. Evaluation of health research networks frequently relies on selected individual methods such as SNA, bibliometric evaluation, and membership surveys. However, a more holistic mixed-method approach, using complementary qualitative and quantitative methods, is required to fully address the benefits associated with a health research network from multiple perspectives. The use of established frameworks, the State of the Network Evaluation, and the CAHS framework permits the evaluation of network connectivity, health, and results as a part of the analysis (Panel on Return on Investment in Health Research 2009; Network Impact and Center for Evaluation Innovation 2014). Similar incorporation of network-relevant metrics alongside the CAHS framework has been proposed for the Canadian Institute for Military and Veterans Health Research (CIMVHR) in 2012, encompassing impact subcategories of membership, quality of the network membership, leadership, sustainability, collaboration, network structure, administrative support, and efficiency (Panel on Evaluating CIMVHR 2012). These aspects of network evaluation are incorporated within the State of the Network Evaluation framework. The CIMVHR network evaluation framework proposed by members of the CAHS provides guidance as to the general methods to examine the network's impact. In contrast, our proposed methods provide explicit ways of measuring the indicators within the CAHS framework.

We found, similar to the findings of Alberta Innovates—Health Solutions (AIHS; Graham et al. 2018) that the CAHS framework required modification to meet stakeholder needs and to fully describe the research activities in context to the Network. The adaptation of the CAHS framework was also required, as outlined by the CHSPRA framework for assessing the impact of health services and policy research, to describe the Network activities and influence on health policy and decision making (Canadian Health Services Policy Research Alliance (CHSPRA) Impact Analysis Working Group 2018). We also identified that multiple sources of information are required to capture impact and the need for prospective data collection to complete ROI evaluation. The ability to conduct the network evaluation requires a multidisciplinary team given the diversity of the methods included within the protocol. This includes individuals familiar with SNA, qualitative methods, and bibliometric analyses. In the case of the Diabetes Action Canada SPOR network researchers with these skill sets and resources have been incorporated into the membership to support the longitudinal evaluation of the activities of the network. It permits the ability to prospectively plan and strategically decide when network evaluation is needed, whether for internal quality improvement initiatives or to meet funders and stakeholders’ needs. Further benefits of incorporating the network evaluation team into the network's infrastructure are that data to measure the indicators of ROI for research activities are also incorporated into annual reporting and can also guide research protocol design to include outcome measures relevant to broader determinants of health. Furthermore, the prospective collection of data and analysis throughout the life cycle of the Network allows for reflection of progress in a ‘real-time’ permitting the ability to survey members regarding research primary and secondary outputs, and reduces the challenges associated with retrospective analysis of research impact (Adam et al. 2012).

This network evaluation is intended to be used for several purposes which include: providing information to direct improvement and changes to the Network through presentation and reporting to the Network Steering Council, presentation to the membership, including patient partners, to provide updates on the connectivity, health, and results of the Network, to longitudinally examine the changes to network structure and activities, to report to funders their ROI associated with the grants provided, and finally to develop further methods associated with Network Evaluation.

There are some limitations to this network evaluation approach. It is still faced with the challenges of impact evaluation, including time lag, attribution, knowledge creep, and gathering of evidence (Penfield et al. 2014). Some indicators may not be expected to be fully realized during the life cycle of the network or may be short-lived without long-term impact. It has been suggested that a longer-time horizon be considered to fully examine the ROI on research activities (Adam et al. 2012). The attribution of the impact of the Network activities on the broader health and well-being and economic and societal prosperity will need to be considered. The interpretation of each of the domains and indicators presented in the CAHS framework is summative, and an overall impression of network progress and impact has to be seen by stakeholders. As some of the indicators are based on self-report by the investigators, the actual activities associated with the network may be underreported. In addition, participation by network members may not be equal with a core group of individuals participating in both the network and contributing to the evaluation questionnaires, interviews, and reporting. These limitations in data and reporting will need to be examined within and between evaluations. Comparison of network growth between cross-sectional evaluations of both qualitative and quantitative results will present with challenges. Applying this convergent mixed-methods approach to examine a research network and provide a holistic impression of the Network progress and activities, while of interest to external stakeholders, may also not fully satisfy their evaluation metrics and they may partially rely on other traditional measures that are not included in the current indicators (i.e. i10-index, h-index, journal impact factor). The incorporation of the evaluation team within the network may result in bias in the interpretation of the outputs and results, however, we feel that this is outweighed by the contextual and historical advantage of having the evaluators embedded within the day-to-day participation as network members. Finally, the ability to collect and analyze health network-related activities can be resource-intensive, requires a supportive administrative and governance structure within the network that can oversee data collection and report regularly.

Overall, the approach presented provides the next step in CIHR SPOR Network evaluation and other health research enterprises in Canada. The Network has been funded for another 5-year term, with a restructuring of the network’s programs, aligned with the life cycle of a network, which will provide an opportunity to conduct additional evaluations of the network and examine longer-term impact and ROI of the health research and other activities of the organization. Further the development of methods to fully examine the broader health, economic, and social benefits of Canadian health research activities at a network level is warranted.

## Supplementary data

Supplementary data are available at *Research EvaluationJournal* online.

## Funding

This work was supported by Diabetes Action Canada through funds provided by the Canadian Institutes of Health Research (CIHR) Strategy for Patient-Oriented Research (SPOR) Networks in Chronic Disease.

*Conflict of interest statement*. None declared.

## Supplementary Material

rvac034_Supplementary_Data
