# Supplementary material for: Describing the state of a research network: A mixed methods approach to network evaluation
Source: Res Eval. 2022 Oct 28;32(2):188–99. doi: 10.1093/reseval/rvac034 (PMC10550251; doi:10.1093/reseval/rvac034)
Supplement: rvac034_Supplementary_Data [file rvac034_supplementary_data.docx]

# Interview Guide[[1]](#footnote-2)

## Interview Objectives

1. Describe the perceived purpose of Diabetes Action Canada (DAC) from the member’s perspective;
2. Assess the extent to which DAC met member expectations;
3. Describe outcomes associated with DAC;
4. Identify key success factors for effective interactions and collaboration in DAC; and
5. Identify perceived barriers and/or other factors that limited interactions and collaboration in DAC.

## Interview Guide

I. Introduction

Thank you for agreeing to participate in a telephone interview. My name is **<insert name>**. I am conducting this interview on behalf of **Diabetes Action Canada, also called DAC,** to evaluate network interactions and collaboration as well as the effect of DAC. The purpose of this interview is to help us better understand the effect that the **DAC network** has had on participants, their affiliated organizations, and health care in general.

It is important that you respond to all of the interview questions based on your experience and perspective as a participant in **DAC**. You should have received an email that contained an overview of the types of questions that I would like to ask you today. This email also contained information about the ethics approval for this project such as an explicit consent form. Did you receive that? Do you have any questions before we begin?

II. Interview Questions Related to Objectives #1 and #2

1. From your perspective, what is the main purpose of the **DAC**?
2. Why did you join **DAC**?

*Prompt, as necessary:*

- 1. What did you hope to achieve by joining DAC?
  2. What benefits did you expect to receive as a result of participating in DAC?
  3. When you joined DAC, were you looking for a solution to a specific problem or were you more broadly interested in expanding your knowledge or expertise around the research/clinical topics of DAC?

1. As you became involved in DAC, did you discover other reasons for participating that you did not initially anticipate?
2. In what way(s) has DAC met your expectations and/or needs?
3. In what way(s) has the DAC failed to meet your expectations and/or needs?
4. What could DAC have done differently to meet your expectations and/or needs?
5. How did the network evolve over time?

III. Interview Questions Related to Objective #3

Participation in a research network such as DAC can result in outcomes at many levels, including the individual level, the organizational level, and the higher level at which there is a potential for public health impact (e.g., infrastructure or community health outcomes). We are interested in your perceptions regarding the extent to which Diabetes Action Canada yielded benefits at each of these three levels.

1. Have you been able to use any DAC-created knowledge product(s) (e.g. tutorials, documents, toolkits, guidelines, etc.) developed by DAC in your work to meet a specific need?

*If so, ask interviewee to describe and talk about any additional examples of knowledge products developed by DAC.*

*If respondent indicates that they have been able to use a knowledge product developed by DAC, ask:*

1. In what way(s) do you think the new knowledge products or resources that you have been able to use will benefit individuals, organizations, and public health in general?

*Prompt, as necessary:*

- 1. Do you think you will (or already have) benefitted on an individual or personal level from the new knowledge products or resources? If so, please explain.
  2. Can you think of ways in which your organization will (or already has) benefitted from the new knowledge products or resources? If so, please explain.
  3. Do you think the new knowledge products or resources will have (or already have had) an impact on the health system? If so, please explain.
  4. Do you think the new knowledge products or resources will have (or already have had) an impact on Society? If so, please explain.

1. Has participation in DAC helped you make connections with other people or organizations? If so, what types of connections and with whom?

*Prompt, as necessary:*

- 1. Has participation in DAC helped you make connections with people within your own organization? If so, please describe.
  2. Has participation in DAC helped you make connections with people in other organizations and/or agencies? If so, please describe.

1. In what way(s) do you think your new connections with people or organizations made through DAC have benefitted individuals, organizations, and public health in general?

*Prompt, as necessary:*

- 1. Do you think you have benefitted on an individual or personal level from these new connections? If so, please explain.
     1. Prompt as needed for increased knowledge and expertise, enhanced interpersonal/working relationships, application of knowledge to practice, and improved self confidence/ self efficacy
  2. Can you think of ways in which your organization has benefitted from the new connections made through DAC? If so, please explain.
     1. Prompt as needed for increased individual knowledge that has been shared with others in their home organization, individual knowledge gained in DAC that was applied in their home organization, organization to organization sharing, and efficiencies gained by learning from others
  3. Do you think the new connections have had (or will have) an impact on health care in general, and Diabetes in particular? If so, please explain.
     1. Prompt as needed for changes in health systems, infrastructure, and changes that have allowed the organization to communicate with other organizations and agencies, etc.

1. In your opinion, what are the most important outcomes or benefits that have resulted from DAC?

IV. Interview Questions Related to Objective #4

1. How would you rate the overall success of DAC using a scale from one to ten, where one is a complete failure and ten is a total success?

*Prompt, as necessary:*

- 1. Why?

1. In your opinion, how effective do you think DAC has been in the following[[2]](#footnote-3):
2. Creating a safe environment for the generating and sharing of knowledge and expertise
3. Building a sense of trust among the DAC members
4. Making DAC members aware of the knowledge, skills, and expertise of other members of the network
5. Improving information flow and knowledge reuse among DAC members and their respective organizations
6. Encouraging DAC members to engage in higher-level thinking through grant writing, attending workshops or teaching
7. Developing the capacity of members to engage in collaborative problem solving
8. Improving relationships between other partners like the industry or the Government
9. Enabling the application of knowledge to practice
10. Are there other factors or circumstances that you think contributed to the success (or failure) of DAC? Please explain.

V. Interview Questions Related to Objective #5

1. Did you experience any challenges or barriers (e.g. competing priorities, organizational challenges, job role changes, technological challenges) that kept you from participating in DAC at the level that you would have liked to participate?

*Prompt, as necessary:*

- 1. Were you able to participate in as many of the DAC events as you wanted to participate in? If not, why?

1. Can you think of anything DAC could do differently to address the challenges or barriers that might keep people from participating fully?

VI. CONCLUSION

Those are all the questions I have for you today.

1. Are there any other comments you would like to provide?

Thank you very much for your time.

*<END INTERVIEW>*

1. This interview guide is a close adaptation of the CDC Evaluation Interview Guide to be used to evaluate a community’s impact on the individual members, their organizations, and the public health focus of the community. [↑](#footnote-ref-2)
2. [↑](#footnote-ref-3)
